# Evaluation of selected aspects of the Nutrition Therapeutic Programme offered to HIV-positive women of child-bearing age in Western Cape Province, South Africa

**DOI:** 10.4102/sajhivmed.v16i1.338

**Published:** 2015-04-28

**Authors:** Tine T. Hansen, Marietjie Herselman, Lisanne du Plessis, Luzette Daniels, Tirsa Bezuidenhout, Cora van Niekerk, Laura Truter, Per O. Iversen

**Affiliations:** 1 Division of Human Nutrition, Stellenbosch University, Tygerberg Campus, South Africa; 2 Department of Nutrition, Institute of Basic Medical Sciences, University of Oslo, Norway

## Abstract

**Background:**

The Nutrition Therapeutic Programme (NTP) involves the provision of food supplements at primary health clinics (PHCs) to correct nutritional deficiencies in vulnerable groups. Although previous studies have identified problems with implementing the programme at PHCs, assessments of its efficiency have been scarce.

**Objective:**

To evaluate implementation of the NTP at PHCs that provide antiretroviral therapy.

**Methods:**

A cross-sectional, descriptive study was conducted at 17 PHCs located within 3 districts of Western Cape Province. Two target groups were chosen: 32 staff members working at the sites and 21 women of child-bearing age enrolled in the NTP. Questionnaires were used to obtain data.

**Results:**

Only 2 women (10%) lived in food-secure households; the rest were either at risk of hunger (29%) or classified as hungry (61%). Most of the women knew they had to take the supplements to improve their nutritional status, but the majority only recalled receiving basic nutritional advice, and the information was mainly given verbally. Ten of the women had shared their supplements with others, mostly with their children. The study identified lack of clearly defined NTP responsibilities at the PHCs, causing confusion amongst the staff. Although many staff members expressed problems with the NTP, only 38% of them reported having routine evaluations regarding the programme.

**Conclusion:**

Several aspects compromised the effectiveness of the NTP, including socio-economic factors leading to clients' non-compliance. The strategic organisation and implementation of the NTP varied between different PHCs offering antiretroviral therapy, and staff experienced difficulties with the logistics of the programme.

## Introduction

In 1995, the South African government implemented the Integrated Nutrition Programme (INP) as a response to the poor nutritional status of the country's population. A targeted supplementary feeding programme termed the Nutrition Therapeutic Programme (NTP), as one component of the INP, involves the provision of food supplements at primary health clinics (PHCs) to correct nutritional deficiencies in vulnerable groups.^1^ These groups include babies and children (0–18 years), pregnant and lactating women, and patients diagnosed with HIV/Tuberculosis (TB) and other chronic diseases (Table 1). Eligible adult patients are referred and admitted to the NTP based on the following criteria: body mass index (BMI) < 18.5 kg/m^2^; unintentional weight loss > 10% over 6 months and unintentional weight loss > 5% in one month. After 6 months, patients are re-evaluated and, if they suit the exit criteria, they are taken off the programme.^2^ However, studies have identified problems with implementing the NTP at PHCs, including insufficient financing, unclear responsibilities amongst staff, and knowledge gaps amongst the clients.^3,4,5^

**TABLE 1:** Current target groups, entry criteria and product options available for patients on the Nutrition Therapeutic Programme in Western Cape Province.

Target group	Entry criteria	Product
Birth < 6 months	Non-breastfed (medically/mentally unfit mother, mother on long-term medication/treatment contra-indicated for breast feeding or mother died)	Infant formula **ONLY** – whey dominant
6–12 months	Growth faltering or mid upper arm circumference (MUAC) = < 125 mm	Infant formula whey dominant **AND** maize-based infant cereal
1–13 years	Growth faltering or MUAC: 1–5 years = < 135 mm 6–9 years = < 155 mm 10–14 years = < 185 mm	Maize-based instant porridge **AND** lactose-free energy drink **OR** maize-based Instant porridge AND lactose-free energy drink.
		Lactose-free energy drink **ONLY**
		Maize-based instant porridge **AND** RuTF
14–18 years	Growth faltering or MUAC (5–14 years)	Maize-based instant porridge **AND** lactose-free energy drink
		Maize-based instant porridge **AND** RuTF
		Lactose-free energy drink **ONLY**
Severe acute malnutrition (SAM) children: birth ≤ 5 years	On ‘old’ Road-to-Health chart: < 60% expected weight-for-age.	**Infant: 0 – 12 months:** 0–6 months: infant formula **ONLY:** 6–12 months: infant formula **AND** rice/mealie-meal-based infant cereal **Children 1–5 years:** Maize-based instant porridge **AND** lactose-free supplementary drink
	On ‘new’ Road-to-Health booklet: -3SD for weight-for-age or weight-for-height MUAC (1–5 years)	
Pregnant women and lactating women (infant < 6 months)	Insufficient growth of fetus (symphysis-fundus graph) MUAC < 23 cm	Lactose-free energy drink (with/without fibre) only **OR** maize-based instant porridge **AND** lactose-free energy drink
	Lactating women with/without growth-faltering infant (< 6 months) and/or MUAC < 23 cm or BMI < 18.5	Maize-based instant porridge **AND** RuTF
Lactating women (infant > 6 months)	Lactating women with/without growth-faltering infant (> 6 months) MUAC < 23cm or BMI < 18.5	**Mother:** Lactose-free energy drink only **OR** maize-based instant porridge **AND** lactose-free energy drink
		Maize-based instant porridge **AND** RuTF
		Infant: infant cereal
HIV and AIDS and TB and other chronic diseases	Birth – 60 months: Growth faltering or referred by dietician	> 18 years: Maize-based instant porridge **AND** lactose-free energy drink **OR** maize-based instant porridge **AND** RuTF drink
	5–18 years: growth faltering or referred by dietician	Maize-based instant porridge **AND** RuTF drink
> 18 years – BMI < 18.5 **OR** > 10% unintentional weight loss in 6 months **OR** > 5% unintentional weight loss in past month	Lactose-free energy drink only
		Maize-based instant porridge **AND** RuTF
		RuTF only

MUAC, mid upper arm circumference; BMI, body mass index; RuTF, ready-to-use therapeutic food.

South Africa is severely affected by HIV. Even though antiretroviral therapy (ART) coverage increases, the government still faces challenges in combating the spread of the disease. Good nutrition is crucial when infected with HIV, as both the disease itself and the treatment have metabolic consequences.^6,7^ Also, a lowered BMI, which is associated with malnutrition, might increase mortality in HIV patients.^8^

The present study evaluated the NTP at PHCs offering ART, with particular focus on women, as this group had previously not been addressed.^3,4,5^ As the NTP relies largely on compliance of the individuals receiving the supplements, it is important to understand how well the programme is implemented at clinic level and adhered to at household level, to ensure that the maximum number of patients reap the full NTP benefits. Therefore the main aim of the study was to establish the opinions, perceptions and current practices of HIV-positive female beneficiaries of the NTP. It was further necessary to investigate whether patients received adequate information and education regarding the NTP to utilise it effectively. Lastly, we also examined the opinions, perceptions and practices of staff members concerning the NTP and the resources at their disposal for effectively implementing and managing the programme.

## Methods

A cross-sectional descriptive study was conducted in the City of Cape Town, Overberg and Cape Winelands districts from December 2009 to April 2010, and from March 2011 to February 2012. Ethical approval was obtained from the Health Research Ethics Committee at Stellenbosch University (ref no: N07/10/232), the Provincial Government of the Western Cape, the Municipality of the City of Cape Town, and the Norwegian Regional Committee for Medical and Health Research Ethics (ref. no. 2009/1364b).

### Study sites and participants

Of the 52 registered PHCs that provide ART, 17 were randomly included. Twelve were within the City of Cape Town, and 5 in rural areas of the Cape Winelands and Overberg districts. Data obtained from urban and rural sites were pooled for analyses.

We targeted two groups: (1) various staff members (managers, nurses, dieticians) at the PHCs working with the NTP and (2) female clients (15–49 years old) enrolled in the NTP. Thirty-two staff members were included, with all PHCs represented. Clients were selected by convenience sampling as randomisation was not feasible. Clients eligible for inclusion were either approached on the day of a clinic visit, or identified by means of the NTP register. Twenty-one women were included from 10 of the sites, whilst no women were eligible at the remaining 7 sites.

### Data collection methods

Questionnaires adapted from a previous study regarding the NTP conducted at PHCs were used.^4^ The questionnaires for both clients and staff members were available in Afrikaans, English and isiXhosa. Clients' experience with and knowledge about the NTP were collected through an interviewer-administered questionnaire containing both option-based as well as open questions. A validated hunger questionnaire was used as a proxy for food insecurity experienced in clients' households and was based on 8 questions addressing the availability of food and their ability to secure an adequate food intake.^9^ Staff members were questioned about their training, knowledge, opinions, perceptions, practices and resources regarding the NTP, also through both open and option-based questions. In addition, sociodemographic data were obtained.

## Results

### Characteristics of the clients

The median age of the 21 clients included was 30 (range 16–46) years. Seventeen of them received ART, 5 had AIDS and 17 had TB.

Most of the women were unemployed (86%), and only 33% had grade 11–12 education or higher. Table 2 shows the income and money spent on food per household. Twelve (57%) of the women stated that they and/or their family received social grants, including child support (58%), old age pension (25%), disability grant (8%), or financial support from other family members (17%).

**TABLE 2:** Income and food expenses per household.

Income and expenses	Rands†	Number of clients	
		n	%
Monthly income	Nothing	3	14
	1–500	6	28
	501–1000	3	14
	1001–3000	6	28
	3001–5000	2	10
	Do not know	1	5
Weekly food expenses	0–100	6	28
	101–200	7	33
	201–400	3	14
	> 400	2	10
	Do not know	3	14

†, 1 Rand ~ 0.09 US dollars ~ 0.07 Euro.

### Food insecurity

Scores from the hunger questionnaire was used to identify household food insecurity. Thirteen out of the 21 women were classified within the *hungry* category. Four of these 13 women answered Yes to all 8 questions, indicating severe household food insecurity. Six women were *at risk of hunger*, and only 2 had scores that put them in the *food secure* category.

### Clients' experiences of the Nutrition Therapeutic Programme

When clients were asked if they knew why they were receiving supplements, 19/21 (90%) answered Yes. Only 2 women said that they did not follow instructions given by staff members for use of the supplements.

Fourteen reported receiving nutritional advice before receiving supplements. Sixteen received verbal instructions on how to take the supplements and/or information as to why they had to take them. Only 4 had received any written information about the NTP and supplements, whilst 5 said that they had not received any information. Two women had experienced problems with the supplements owing to a lack of ingredients to mix them with. Regarding side-effects, 5 women reported nausea, but 3 of them specified that this occurred only when starting to use the supplements. None of the responders reported that they had ever sold their supplements, but 10 had shared the supplements with others, mainly their children.

### Staff members' characteristics

The positions of the 32 staff members (30 female and 2 male) were as follows: 8 facility managers, 10 professional nurses, 5 dieticians, 3 nursing staff, 2 medical doctors and 4 defined as *other* (2 health promoters, 1 nutrition adviser and 1 nutritionist). The median age of the staff members was 40 (range 26–56) years.

### Knowledge, training and responsibilities of staff members regarding the Nutrition Therapeutic Programme

Twenty of the staff members had received training and/or education in implementing the NTP after their formal training, including oral lecture (65%), written material (45%) and practical demonstration (45%). Thirteen had received training once, whilst only 7 had regular training.

When asked what role staff had in the NTP, the categories most frequently answered were *Handing out supplements* (66%) and *Recording statistics* (66%), followed by *Storage* (44%) and *Ordering* (34%). Regarding storage, 71% of the staff with this responsibility had written guidelines on how to store the supplements, but 43% reported that their facility was inadequate because of space constraints. For handing out supplements, 90% with this responsibility had written instructions. All staff members indicated that they explained to clients the purpose of the supplements, but only 29% reported giving clients written information.

Staff found that clients receiving supplements were generally grateful. One of the stated reasons for poor compliance was clients failing to come back for follow-up appointments. Three staff members said this was because of ignorance; the rest thought that social factors (e.g. no funds for transport to the clinic, or important activities such as work) prohibited clients from attending scheduled appointments.

When asked to specify who were in charge of the different components of the NTP, staff members gave inconsistent answers (Table 3). There was a discrepancy between sites regarding which staff categories were assigned NTP responsibilities, but answers also differed between staff members working at the same site.

**TABLE 3:** Distribution of Nutrition Therapeutic Programme responsibilities amongst primary health clinics site staff categories.

Staff category	Ordering supplements	Storage of supplements	Deciding entry/exit of NTP	Handing out supplements	Updating NTP register	Statistics and reporting
Facility manager	28	22	3	0	0	13
Professional nurse	25	34	50	47	47	50
Nursing staff	0	0	25	28	16	9
Pharmacist	6	19	0	6	0	0
Dietician	38	25	50	44	41	34
Others	16	22	22	25	13	6
Do not know	3	6	0	3	3	9

Note: Values refer to which staff categories the 32 staff members allotted the various responsibilities, and are expressed as percentages. For example, 28% of the 32 staff members answered that the facility manager was responsible for ordering the supplements. It was possible to allocate several staff categories for each area of responsibility.

NTP, Nutrition Therapeutic Programme.

Nineteen staff members stated that there was a lack of supplements within the last year. Reasons for this were mainly lack of stock in the clinic (32%) or lack of stock at the suppliers (63%). Five staff members said that the clinic's budget had been exceeded. Eight staff members said that they had tried to improve the nutrition budget with their superiors; 5 had succeeded. Twelve of the 32 staff members stated that their clinic had undergone evaluations of their NTP implementation routines. When asked to specify what problems they experienced with the NTP, 2 commented that a staff shortage was an issue, and said that staff had inadequate time to implement the programme properly. Five staff members said that inadequate clinic routines prohibited efficient utilisation of the NTP, and another 5 staff members attributed lack of success with the programme to clients' failing to come back for follow-up appointments.

Six of the 8 facility managers said that their staff had received training in implementing the NTP. Only 2 managers reported that the training took place regularly; this corresponded well with what staff members had responded about training. Seven facility managers had a separate budget for the NTP, and 2 stated that their budget was never adequate to cover expenses related to the NTP.

## Discussion

Our results indicated that there were several aspects that potentially compromised the effectiveness of the NTP, including socio-economic factors leading to clients' non-compliance. The strategic organisation and implementation of the NTP varied between clinics, and staff experienced some difficulties with the programme logistics at PHCs.

Many of the women studied experienced food insecurity; this jeopardised their nutritional status and increased the need for supplementation, which could result in clients becoming dependent on supplements. In theory, these women should benefit from the supplements, but it was evident that many shared their supplements with others, indicating a shortfall in taking the advised dosage. This conclusion was further supported by findings from the hunger questionnaire that established that more patients reported reducing the size of their own meals rather than those of their children.

The long-term effectiveness of the programme depends largely on the ability of former clients to sustain nutritional status after they have been exited from the NTP. This is difficult for them as they have increased nutritional needs owing to HIV and AIDS and TB, and the task is made even more challenging when their socio-economic status is low. Malnutrition also hastens disease progression amongst such people and puts added pressure on healthcare services.

Educational levels affected the understanding and effective utilisation of supplementation amongst clients. Even though the women understood that they had to take the supplements to gain weight, more in-depth counselling, or use of illustrated instructions and/or written information in their own language, could have contributed to a better understanding of the programme and possibly promoted better adherence. Both the clients and staff experienced difficulties in implementing the programme. One of the main problems identified by staff members was poor compliance of the women in attending clinic. If client attendance reflects reality, it can be assumed that clients failing to attend follow-up appointments have increased the defaulter rate of the programme. It must be noted that client compliance was largely influenced by their socio-economic difficulties, which manifest as poor clinic attendance, despite the fact that most of the attending clients were perceived to be grateful for the supplements and support they received.

The lack of strategic organisation, and no clear responsibility distribution at clinic level, must be suspected of causing obstacles to efficiency and programme coverage. The scarcity of evaluation routines would indicate that any problems experienced with the programme would continue without being properly addressed; an example is the lack of supplements owing to poor ordering routines, which could easily be corrected.

Considering South Africa's burdens of malnutrition, the lack of food and nutrition security and the need for multisectoral interventions, the low health sector budgetary allocation by government to nutrition (less than 0.3% of the health budget) is a further problem. In current expenditure, most funds are spent on targeted supplementary feeding, including to HIV-infected individuals as a target group. Appropriate monitoring and evaluation of this programme is therefore of cardinal importance. There is a concern that the returns on nutrition expenditure are not as high as could be, owing to inappropriate interventions and poor implementation.^10^

The National Nutrition Directorate (NND) acknowledges the challenges that the programme faces, including: poor institutional co-ordination amongst various departments that are giving assistance to people facing food and nutritional insecurity; lack of proper mechanisms to facilitate referral of individuals across programmes; under-developed information management; different entry and exit criteria for nutritional support for people living with HIV and AIDS and TB; and little or no community involvement in nutrition support interventions for such people.^10^ However, very little strategic guidance from the NND is provided to overcome these challenges.

### Limitations

The main limitation of this study was the small number of eligible clients identified at the PHCs. The reason for the specific inclusion criteria was because these individuals form part of the vulnerable group in society of whom very little is known. Although most clinics had specific days on which the NTP supplements were handed out, and researchers made a concerted effort to remind them of their appointments, this did not ensure that clients would visit the clinic on those days. Despite the small number of participants, the data obtained were extensive. Through open questions, clients could freely express opinions and experiences, and thus provided us with a good understanding of whether they felt the programme worked optimally, both for staff members issuing supplements, and also for the clients receiving them. Notably, the study was performed in the Western Cape – one of the most affluent provinces in the country, and this fact might limit the generalisability of our findings.

## Conclusion

Our results support the findings from previous studies that there are problems with the implementation of the NTP and that these problems are also evident at PHCs.^1,3,4^ It is clear that the challenges experienced by staff members and clients are obstructing the effective implementation of the programme. It is therefore necessary to take note of the relevant challenges mentioned in this study and strive to solve the problems that, if left unattended, could ultimately result in clients not reaping the benefits of the programme, which in turn will lead to increased government expenditure and depletion of clinic funds in support of a programme that is ineffective, as patients are not reaching the goals set out in the programme. Therefore, to successfully implement nutrition interventions, sufficient capacity and resources, strategic management, evaluation routines and monitoring of results must be in place.^11,12,13^ The findings from our study advocate for an increased effort in these matters when implementing the NTP at PHCs that provide ART in Western Cape Province and possibly also elsewhere in South Africa.
